# Paradigm Shift in Antimicrobial Resistance Pattern of Bacterial Isolates during the COVID-19 Pandemic

**DOI:** 10.3390/antibiotics10080954

**Published:** 2021-08-07

**Authors:** Vikas Saini, Charu Jain, Narendra Pal Singh, Ahmad Alsulimani, Chhavi Gupta, Sajad Ahmad Dar, Shafiul Haque, Shukla Das

**Affiliations:** 1 Department of Microbiology, University College of Medical Sciences & GTB Hospital, Delhi 110095, India; vikassaini287@gmail.com (V.S.); doccharujain@gmail.com (C.J.); singhanjna@yahoo.co.in (N.P.S.); sdar@jazanu.edu.sa (S.A.D.); 2 Medical Laboratory Technology Department, College of Applied Medical Sciences, Jazan University, Jazan 45142, Saudi Arabia; ahmad.alsulimani@hotmail.com; 3 Northern Railways Central Hospital, New Delhi 110055, India; chhavi13.86@gmail.com; 4 Research and Scientific Studies Unit, College of Nursing, Jazan University, Jazan 45142, Saudi Arabia; 5 Faculty of Medicine, Görükle Campus, Bursa Uludağ University, Nilüfer 16059, Turkey

**Keywords:** antibiotics, antimicrobial resistance, bacterial co-infection, COVID-19, irrational consumption

## Abstract

Antimicrobial resistance (AMR) is an emerging public health problem in modern times and the current COVID-19 pandemic has further exaggerated this problem. Due to bacterial co-infection in COVID-19 cases, an irrational consumption of antibiotics has occurred during the pandemic. This study aimed to observe the COVID-19 patients hospitalized from 1 March 2019 to 31 December 2020 and to evaluate the AMR pattern of bacterial agents isolated. This was a single-center study comprising 494 bacterial isolates (blood and urine) that were obtained from patients with SARS-CoV-2 admitted to the ICU and investigated in the Department of Microbiology of a tertiary care hospital in Delhi, India. Out of the total bacterial isolates, 55.46% were gram negative and 44.53% were gram positive pathogens. Of the blood samples processed, the most common isolates were CoNS (Coagulase Negative *Staphylococcus*) and *Staphylococcus aureus*. Amongst the urinary isolates, most common pathogens were *Escherichia coli* and *Staphylococcus aureus*. A total of 60% MRSA was observed in urine and blood isolates. Up to 40% increase in AMR was observed amongst these isolates obtained during COVID-19 period compared to pre-COVID-19 times. The overuse of antibiotics gave abundant opportunity for the bacterial pathogens to gradually develop mechanisms and to acquire resistance. Since the dynamics of SARS-COV-2 are unpredictable, a compromise on hospital antibiotic policy may ultimately escalate the burden of drug resistant pathogens in hospitals. A shortage of trained staff during COVID-19 pandemic renders it impossible to maintain these records in places where the entire hospital staff is struggling to save lives. This study highlights the extensive rise in the use of antibiotics for respiratory illness due to COVID-19 compared to antibiotic use prior to COVID-19 in ICUs. The regular prescription audit followed by a constant surveillance of hospital infection control practices by the dedicated teams and training of clinicians can improve the quality of medications in the long run and help to fight the menace of AMR.

## 1. Introduction

Coronavirus disease 2019 (COVID-19) had an immense worldwide impact on health care systems, economy and financial resources. The dynamics of COVID-19 disease and its treatment has been changing throughout the course of pandemic with new research guidelines constantly being added. Steroids, undeniably, are the mainstay in the management of COVID-19 illness, particularly in hypoxemic patients. There has been an irrational use of steroids in mild to moderate symptoms, thus, providing a niche for impending secondary infections. The COVID-19 illness has a wide spectrum of presentation, with a predominance of respiratory distress or fever and sore throat or may even remain asymptomatic. The development of sepsis with raised CRP is generally considered to be a domain of bacteria associated infection but, during this pandemic, viral sepsis emerged as a new entity that is relatively less suspected and undiagnosed, with a remarkable high risk of multiple organ failure [1]. Viral agents causing severe disease are frequently associated with immunosuppression, predisposing patients to secondary infections that can further complicate the course of the disease by increasing the morbidity and mortality. Overlapping clinical features, radiological findings and laboratory parameters renders it difficult to distinguish severe COVID-19 infection from secondary bacterial or fungal involvement. In such clinical uncertainties, in order to salvage the patient, clinicians often preferred a multi-pronged approach, including the use of broad-spectrum antibiotics [2,3]. Furthermore, during the pandemic, country wide attention of microbiologists was drawn towards setting up of molecular laboratories for detecting SARS-CoV-2. A similar urgency was expected in hospital preparedness for admitting critical COVID-19 patients preparation. The COVID-19 pandemic, thus, significantly affected the health services of the country. The surge of patients in the hospitals, lack of paramedical staff and non-availability of facilities, such as ventilators and oxygen supply, inadvertently disrupted several essential programs, such as national health programs, antimicrobial stewardship and hospital infection control measures [4]. Consequently, the world witnessed the emergence of multidrug-resistant superbugs paving the path to another pandemic of antimicrobial resistance and rise of health care associated infections [5,6]. During the pre-pandemic period, antimicrobial resistance (AMR) was the world’s most important health crisis; however, with the emergence of SARS-CoV-2 all efforts were focused on COVID-19 and its management, resulting in a certain degree of compromise on antibiotic usage and audit [7,8]. The exact prevalence of COVID-19 with bacterial co-infection is unknown, further potentiating unnecessary empirical antibiotic coverage [9,10,11,12]. Initial data reported 50% death in COVID-19 patients due to secondary bacterial infections [2]. Later, substantial data suggested low rates of secondary bacterial infections in COVID-19 disease with a meta-analysis of hospitalized COVID-19 patients reporting only 8.02% bacterial co-infection, while others studies demonstrated none [9,13,14].

It is yet to be ascertained whether the majority of severe COVID-19 hospitalized patients received any major benefits with the administration of antibiotics. In order to intensify the conflict, clinical evidence suggest that inadequate broad-spectrum empiric antibiotic use could be associated with higher mortality, especially in case of sepsis [15]. Such differing assumptions will eventually result in an unprecedented emergence of AMR and compromise the global efforts to combat AMR [16,17,18,19,20,21]. Hence, the World Health Organization (WHO) raised concerns regarding the irrational consumption of antibiotics during the COVID-19 pandemic and strictly prohibited the antibiotic prophylaxis or treatment of the patients with mild or moderate COVID-19 unless indicated [16].

In this study, we aimed to evaluate the AMR pattern of bacterial agents isolated from clinical samples (blood and urine) submitted to Microbiology laboratory from the patients with COVID-19 illness, to evaluate those admitted to a tertiary care center during the pandemic and compared the anti-microbial susceptibility profile of bacterial agents isolated in the previous year (pre-COVID-19) for strengthening the antimicrobial stewardship protocols during the disaster.

## 2. Results

The data were analyzed in two time periods: pre-COVID-19 (March 2019 to December 2019) and the COVID-19 period (March 2020 to December 2020).

### Distribution of Isolates

The total number of samples received during pre-pandemic-period (7309) was compared to the (4968) samples received during the pandemic phase in 2020. A decline in bacterial growth was observed during the pandemic (9.94 vs. 11.54%). A total of 494 bacterial isolates (blood and urine) were obtained from patients with SARS-CoV-2 admitted to the ICU of which 55.46% were gram negative and 44.53% were gram positive pathogens. The distribution of isolates among age groups did not show any significant variation during both time frames except in less than 18 years of age, including infants. A male preponderance was observed amongst the clinical isolates obtained during the COVID times compared to pre-COVID-19 period. There was a statistically significant increase in demand for culture requisition from the ICU during the COVID-19 period (63.36%) compared to previous years (5.68%).

There was a significant increase in the proportion of blood culture samples (67.87%) received as compared to urine culture (32.12%) during the pandemic. However, blood culture positivity was lower (7.53%) during the pandemic period compared to the previous year (15.45%). The percentage of urinary bacterial isolates (15.03%) was much higher in samples received from Covid-19 patients in 2020 compared to positivity (8.30%) in the previous year (Table 1).

In Table 2 we observed a similar pattern of distribution of organisms in blood culture, namely *Staphylococcus aureus*, Coagulase Negative *Staphylococcus* (CoNS) and *Enterococcus* spp. during both periods. Among the Gram negative bacteria, even though the positivity rate was similar, there was an increase in the isolation of *Acinetobacter baumannii* and *Escherichia coli* during the pandemic. There was significant increase in urinary isolates detected during the COVID period (82.08% vs. 69.27%).

In the antimicrobial susceptibility pattern of blood culture isolates, ciprofloxacin and cotrimoxazole showed reduced susceptibility among the Gram negative bacteria (Table 3). *Klebsiella pneumoniae* was less susceptible to aminoglycosides, fluoroquinolones and carbapenems. On the other hand, *Pseudomonas aeruginosa* isolates showed alarming resistance to imipenem along with third generation cephalosporins.

*Staphylococcus aureus* depicted reduced susceptibility to all classes of drug during the pandemic period. Significant increases in MRSA during the COVID-19 period with decreases in susceptibility to commonly prescribed drugs such as clindamycin, erythromycin and fluroquinolones were observed as shown in Table 4.

It was observed that *Escherichia coli* and *Acinetobacter baumannii* showed increased resistance to most of the drugs, while the susceptibility pattern for *Klebsiella pneumoniae* did not vary in both periods. Although *Pseudomonas aeruginosa* isolates were less in number, the resistance patterns for various antibiotics were not significantly different except for piperacillin-tazobactum and cefepime (Table 5).

Amongst the urinary isolates, the susceptibility of *Staphylococcus aureus* to erythromycin was reduced in the COVID-19 period than the previous year (Table 6).

## 3. Discussion

The COVID-19 pandemic has brought not only serious economic downfall but bitter challenges in health care settings. The world witnessed the uncontrolled use of broad-spectrum antimicrobials with multiple drug combination regimes administered to patients admitted to wards and in ICUs. Under massive criticism and administrative pressure, a panic-like situation created an abysmal failure in patient management during the pandemic. The fear of acquiring COVID-19 infection and hesitancy to enter COVID-19 ICU and wards amongst health workers resulted in complete collapse and unintentional neglect in health care system, which eventually compromised the established program of antimicrobial stewardship policy. Over the counter availability of drugs, indiscriminate use of steroids and multiple doctor shopping further worsened the situation to the extent that patients with moderate symptoms received multiple courses of antibiotics. Furthermore, the inability to demarcate viral and bacterial associated respiratory complications; poor turnaround time of culture reports and the severity of manifestation among patients were the few underlying factors, which prompted clinicians to start pre-emptive antibiotic. Studies have reported 3.1–3.5% of bacterial co-infection in COVID-19 patients, while 15% secondary bacterial infections after hospitalization are reported under AMR surveillance in India, although mortality was as high as 56.7% in these patients compared to 10.6% in total COVID-19 patients [22]. Secondary infections in admitted patients are known to be associated with negative health outcomes and worsening of the clinical prognosis. In the present pandemic, the situation was similar to a double-edged sword with empirical antibiotics recommended but unaided by the local antimicrobial susceptibility pattern, resulting in increased antimicrobial resistance among nosocomial pathogens [9,13,14]. A comprehensive study by Khurana et al. observed 13% secondary bacterial infection and 84% increase in AMR pattern for bacterial isolates in COVID-19 patients admitted to the hospital [23]. In our study, we observed 7.53% of bloodstream infection (BSI) and 15.03% urinary tract infection (UTI) in COVID-19 patients with higher rates in those admitted to intensive critical care. Similar findings with 6.9% of bacterial co-infections in COVID-19 was also reported by Strathdee et al. from USA [24].

Our 1800 bed tertiary care hospital observed an exponential rise in the number of cases with moderate to severe symptoms of COVID-19 during the pandemic. The customary blood culture tests are essential for the diagnosis of any acute febrile illness. Although these tests are expected in cases with suspected bacterial etiology, COVID-19 pandemic caused a surge in the number of requests for blood culture in many setups including ours. Surprisingly, the number of positivity of blood cultures in COVID-19 patients was found to be low despite a concurrent rise in requests. Similar findings were reported by Sepulveda J et al., with almost 38% increase in the demand for blood culture [25]. Unlike in patients with severe influenza, a higher prevalence of bacteremia was observed while others reported lower rates of bacteremia in patients with other respiratory viral infections, such as respiratory syncytial virus (RSV) including SARS-CoV. [26,27,28].In the realm of viral sepsis, the exact reason for lower bacteremia rates in COVID-19 patients perhaps remains uncertain, as is depicted in negative in blood culture reports [24].

Furthermore, we observed a predominance of Gram positive bacteria during the pandemic, although the pattern did not differ from the previous years. This is in concordance to other studies by David Yu [29] and Cultrera R [30]. The most common amongst the Gram positive bacteria was CoNS followed by *S. aureus* and *Enterococcus* species. MRSA was marginally raised from pre-COVID-19 times although multidrug resistant CoNS were found to be high during the COVID-19 period. [31,32,33]. Several other studies also reported a high proportion of CoNS and *Staphylococcus aureus* as the most common agent isolated from the blood culture of COVID-19 patients [25,30]. CoNS, which are skin commensals, may be considered as contaminants in blood cultures and its role as pathogen is difficult to establish. However, the predominance of CoNS has been increasingly reported as a blood stream pathogen, particularly in immunocompromised settings [31]. The prolonged hospital stay with increased use of intravascular devices, indwelling catheters, use of steroids and biologics and underlying dysregulated immune system with failure of bundled care for preventing device associated infections could be amongst the several underlying reasons for witnessing a rise in CoNS bloodstream infections [25,31,32,33,34,35].

The *Staphylococcus* species showed low sensitivity to erythromycin and ciprofloxacin. This in concordance with the Indian Network for Surveillance of Antimicrobial Resistance (INSAR) data, which reported a high rate of resistance to ciprofloxacin, gentamicin, cotrimoxazole, erythromycin and clindamycin. On the contrary, among Gram negative bacteria, Enterobacteriaceae as a group accounted for maximum number of cases (20–21%) similar to findings reported in other studies [36].

Amongst the Gram negative bacteria, *Acinetobacter baumannii* was the predominant bacteria isolated during COVID-19 compared to the pre–COVID-19 period with reduced susceptibility to gentamicin, amikacin and ciprofloxacin but an alarming decline in susceptibility was observed for Cotrimoxazole and piperacillin-tazobactam. Before the pandemic, *Klebsiella pnemoniae*, followed by *Acinetobacter* spp., *Escherichia coli* and *Pseudomonas aeruginosa* were the common isolates in blood culture. The COVID-19 period witnessed a shift towards *Acinetobacter baumannii* emerging as the most predominant pathogen followed by *Escherichia coli* and *Klebsiella pneumonia*. *Acinetobacter baumannii* associated VAP (ventilator-associated pneumonia) and associated secondary bacteraemia among patients in ICUs contribute to high mortality rates up to 50% [37,38]. *Escherichia coli* is also responsible for community-acquired pneumonia with 11–21% mortality and a disproportionally high rate of bacteraemia [39].

Several factors such as the overuse of cephalosporins and fluroquinolones for respiratory illness in admitted patients as well as for outpatients accounted for the development of a high degree of resistance amongst this class of antimicrobials. Additionally, the rise of Carbapenem resistant Enterobacteriaceae (CRE), CRPA and CRAB pose serious challenges in the management of critically ill patients in all health care settings. Therefore, utmost caution should be advocated, especially in the use of reserve drugs such as amikacin, polymyxin and Tigecycline and combinations for managing bacterial infections in COVID-19 patients [40,41,42]. According to a meta-analysis by Rawson et al., 8% of patients were reported as experiencing bacterial/fungal co-infection during hospital admission [12]. Supported by many researchers, the presence of co-infection manifests unfavorable outcome in COVID patients. [43,44,45,46].

Amongst the urinary isolates, the culture positivity increased to almost double (15% versus 8.3%) during the pandemic in comparison to previous year. A study by Can O et al. showed that urine tract infections were significantly increased in elderly patients after COVID-19 infection [47,48]. *Escherichia coli* remained the most common pathogen followed by *Enterococcus, Staphylococcus* and *Klebsiella pneumoniae* in the urine samples of the COVID patients. The distribution shifted in 2020 with an increase in the proportion of Gram negative isolates from 69 percent to 82 percent. Similar to blood culture isolates, a low susceptibility was observed for cephalosporins, fluoroquinolones and cotrimoxazolein. Higher susceptibility was observed for aminoglycosides, carbapenems, nitrofurantoin and Fosfomycin; resistance exhibited for fluoroquinolones.

The critical observation of our study was the excessive use of intravenous antimicrobial agents such as fluoroquinolones, cephalosporins, carbapenems, azithromycin, vancomycin and linezolid, which belong to the watch and reserve group of drugs of AWARE classification by the WHO. The trend of increasing antimicrobial resistance observed during the pandemic compared to the preceding pre-COVID-19 year was comparable to observations from Clancy et al. [13], who emphasized that the overuse of antibiotics provides abundant opportunity for the bacterial pathogens to gradually develop mechanisms to acquire drug resistance [6,49,50].

Our study emphasizes that the threat of AMR is large, thus, when faced with difficultly to demarcate whether a COVID-19 patient is progressing to a bacterial infection, a strong clinical suspicion aided with serum biomarkers assays such as PCT, CRP and bacterial cultures should be adopted as potential laboratory tools to guide the clinicians to administer antibiotics rationally [50,51].

The association between AMR and COVID pandemic is slowly emerging with many studies (mostly from Asian countries) showing an unbalanced approach toward antimicrobial consumption [16]. Although the burden of infections might have been low, the world witnessed the excessive use of empirical antibiotics in the very first week of admission setting up the ground for rising drug resistance amongst the bacterial isolates. 

## 4. Conclusions

Perhaps more stringent antimicrobial stewardship protocols in COVID-19 management plans may prevent the AMR emergence. Descriptive data of antimicrobial susceptibility should be available to the clinicians for timely de-escalation and interventions in order to reduce inappropriate prescription of antibiotics for COVID-19 patients. Further studies are required to explore the risk factors in secondary bacterial co-infections in COVID-19 patients and severity should be monitored with serum biomarkers before administering empirical antimicrobials. The regular prescription audit followed by a constant surveillance of hospital infection control practices by the clinical hospital infection control team and the training of clinicians can improve the quality of medications in the long run and to help to fight the menace of AMR.

## 5. Material and Methods

### 5.1. Study Description

This was a single-center study conducted at the Department of Microbiology of a tertiary care hospital in Delhi, India. The hospital catered to COVID-19 suspects/ confirmed patients and was declared as a dedicated COVID-19 hospital by the government of Delhi, India. The study was conducted from 1 March 2019 to 31 December 2020. Bacterial isolates from blood and urine samples submitted for microbiological analysis for admitted patients were included in the study. A total of 844 pre-COVID-19 (Mar 2019 to December 2019) and 494 COVID-19 period (March 2020 to December 2020) bacterial isolates were identified and the antimicrobial susceptibility pattern was studied.

### 5.2. Sample Processing and Isolate Identification

Clinical samples (blood and urine) from patients admitted to ICU and wards of our tertiary care COVID-19 hospital were included in the study. COVID-19 period samples were transported and processed as per standardized laboratory protocols set up for COVID-19 patients.

Patients admitted in the hospital were either suspects, clinically diagnosed COVID-19 or confirmed COVID-19 patients. The decision to request for cultures was based on the individual clinical judgment of the treating physician and no systematic protocol was used. Blood and urine samples from these patients were transported to the department as per COVID-19 related precautions. All samples were handled by trained personal while wearing N95 mask and suitable personal protective equipment in a class-II A2 biosafety cabinet. The amount of 10–20 mL venous blood sample was collected and sent in automated BACTEC aerobic blood culture bottles (Becton Dickinson and Company, Sparks, Maryland, USA). Both adult and pediatric bottles were incubated up to 7 days in automated BACTEC machine. Bottles that were flagged positive by the machine were withdrawn and gram staining was performed. Subcultures were performed on blood agar and MacConkey agar plates were incubated overnight at 37 °C for the isolation of the pathogen. Growth was subjected to the microscopic examination of gram-stained smears prepared from the colonies.

For urine cultures, the samples included midstream clean catch and sample from catheterized patients. Urine routine microscopy and cultures were performed. Cysteine lactose electrolyte deficient media were used for semi quantitative methods by using calibrated loops. This method provides information regarding the number of CFU/mL. All cultures were read after 18 to 24 h of inoculation for bacterial growth. Bacterial isolates obtained were processed by the automated identification method using Microscan WalkAway (Beckman Coulter, CA, USA) [52].

### 5.3. BSI Confirmation

BSI was defined as the presence of at least one positive blood culture from a patient. At least two consecutive positive blood cultures were needed to define BSI due to CoNS or other common skin colonizers (e.g., non-diphtheritic *Corynebacteria*, *Bacillus* spp., *Propionibacterium* spp., etc.) [53].

### 5.4. Antimicrobial Susceptibility Testing

The antimicrobial susceptibility test (AST) was performed by the Kirby Bauer disk diffusion method as per CLSI guidelines [54]. Quality control procedures for microscopy and AST were followed by using ATCC strains (*Staphylococcus aureus* 25923 and *Escherichia coli* 25922). Vancomycin and Daptomycin for *S. aureus* could not be included as MIC could not be performed due to the lack of resources.

Biomedical waste management guidelines for COVID-19 were followed for all laboratory work including double bag use and onsite inactivation of samples. Single isolate was reported from all the samples. Duplicate isolates from the same patient on subsequent testing were excluded from the study.

### 5.5. Statistical Analysis

Statistical analysis was performed using the SAS 9.1 software (SAS Institute, Cary, NC, USA). Categorical variables were described by using frequencies and 95% confidence intervals (CIs). Skewed distribution was described using medians, interquartile ranges and maximums and minimums. The significance threshold was 0.05.

## Figures and Tables

**Table 1:** Distribution of samples (Blood and Urine) during pre-COVID-19 and COVID-19 period.

Distribution of Samples	Pre-COVID-19 Period March 2019 to December 2019	COVID-19 Period March 2020 to December, 2020	*p*-Value
Total samples received	7309	4968	
Total bacterial isolates	844 (11.54%)	494 (9.94%)	
Gender			
Female	523 (61.96%)	252 (51.01%)	0.0001
Male	321 (38.03%)	242 (48.98%)	0.0001
Age group			
Infants (<1 year)	91 (10.78%)	32 (6.47%)	0.0081
1–18 year	248 (29.38%)	132 (26.72%)	0.3151
>18 year	505 (59.83%)	330 (66.80%)	0.0119
Hospital /location			
Intensive care unit (ICU)	48 (5.68%)	313 (63.36%)	0.0001
Ward	796 (94.31%)	181 (36.63%)	0.0001
Blood Culture received	3312 (45.31%)	3372 (67.87%)	
Isolates from Blood culture Ward ICU	512 (15.45%) 331/2153 (15.37%) 181/1159 (15.61%)	254 (7.53%) 189/2510 (7.53%) 65/862 (7.54%)	0.0067 0.0067
Urine culture received	3997 (54.68%)	1596 (32.12%)	
Isolates from Urine culture Ward ICU	332 (8.30%) 311/3751(8.29%) 21/246 (8.53%)	240 (15.03%) 228/1516 (15.03%) 12/80 (15.00%)	0.5874 0.5874

(*p* < 0.05 was considered significant.)

**Table 2:** Distribution of commonly isolated pathogens from blood and urine samples (2019–2020).

Bacteria	Blood			Urine		
	2019 (512)	2020 (254)	*p*-Value	2019 (332)	2020 (240)	*p*-Value
Gram positive	345 (67.38%)	177 (69.68%)	-	102 (30.72%)	43 (17.91%)	-
*Staphylococcus aureus*	82 (16.01%)	46 (18.11%)	0.5921	26 (7.83%)	17 (7.08%)	0.8726
Coagulase negative *Staphylococcus*	246 (48.04%)	118 (46.45%)	0.3143	2 (0.60%)	5 (2.08%)	0.1366
*Enterococcus* spp.	17 (3.32%)	13 (5.11%)	0.3203	74 (22.28%)	21 (8.75%)	0.0001
Gram negative	167 (32.61%)	77 (30.31%)	-	230 (69.27%)	197 (82.08%)	-
*Klebsiella pneumoniae*	81 (15.82%)	13 (5.11%)	0.0001	10 (3.01%)	15 (6.25%)	0.2141
*Acinetobacter baumannii*	53 (10.35%)	31 (12.20%)	0.1961	36 (10.84%)	8 (3.33%)	0.0001
*Pseudomonas aeruginosa*	6 (1.17%)	10 (3.94%)	0.0102	30 (9.03%)	6 (2.5%)	0.0002
*Escherichia coli*	27 (5.27%)	23 (9.05%)	0.0170	154 (46.38%)	168 (70%)	0.0001

(*p* < 0.05 was considered significant).

**Table 3:** Comparison of antimicrobial susceptibility pattern of Gram negative bacteria in blood samples between pre-COVID and COVID period.

Blood	*Escherichia Coli*			*Klebsiella pneumoniae*			*Pseudomonas aeruginosa*			*Acinetobacter baumannii*		
	Pre-COVID-19 Period (27)	COVID-19 Period (23)	*p*-Value	Pre-COVID-19 Period (81)	COVID-19 Period (31)	*p*-Value	Pre-COVID-19 Period (6)	COVID-19 Period (10)	*p*-Value	Pre-COVID-19 Period (53)	COVID-19 Period (13)	*p*-Value
Gentamicin	70	66.6	0.7609	32.3	25	0.3474	65	75	0.1646	29	20	0.1881
Amikacin	76	100	0.0001	31	16.6	0.0307	66	100	0.0001	23	20	0.7310
Cefotaxime	36	11.1	0.0001	3.5	8.3	0.3727	NR	NR	-	10	20	0.0734
Ciprofloxacin	36	33.3	0.3801	36	16.6	0.0037	60	50	0.2007	26	20	0.4010
Imipenem	80	88.8	0.1170	52	25	0.0001	83	75	0.2240	7	20	0.0119
Cotrimoxazole	36	22.2	0.0423	25	16.6	0.2240	NR	NR	-	31	20	0.1042
Piperacillin + Tazobactam	59	55.5	0.6684	11	16.6	0.3083	100	100	1.00	33	20	0.0539
Ceftazidime	NR	NR	-	NR	NR	-	66	25	0.0001	NR	NR	-

NR—Not recommended according to CLSI; *p* < 0.05 was considered significant.

**Table 4:** Comparison of antimicrobial susceptibility pattern of Gram positive bacteria in blood samples between pre-COVID-19 and COVID-19 period.

Blood	*Staphylococcus aureus*			Coagulase Negative *Staphylococcus*			*Enterococcus* sp.		
	Pre-COVID-19 Period (82)	COVID-19 Period (46)	*p*-Value	Pre- COVID-19 Period (246)	COVID-19 Period (118)	*p*-Value	Pre-COVID-19 Period (17)	COVID-19 Period (13)	*p*-Value
Tetracycline	79	55.5	0.0008	57	81.8	0.0004	26	20	0.4010
Linezolid	97	77.7	0.0001	100	100	1.00	96	80	0.0008
Clindamycin	53	38.8	0.0649	43	40	0.7742	NR	NR	-
Cefoxitin	46	33.3	0.0223	15	30	0.0171	NR	NR	-
Erythromycin	35	22.2	0.0596	24	15.4	0.0006	ND	ND	-
Cotrimoxazole	75	55.5	0.0072	59	30.8	0.0001	NR	NR	-
High level gentamicin	NR	NR		NR	NR		100	80	0.0001
Ciprofloxacin	29	22.2	0.3304	25	33.3	0.4432	40	20	0.0032

NR—Not recommended according to CLSI; ND—Not performed as it belonged to either group C or O of CLSI; *p* < 0.05 was considered significant.

**Table 5:** Comparison of antimicrobial susceptibility pattern of Gram negative bacteria in urine samples between pre-COVID-19 and COVID-19 period.

Antibiotics	*Escherichia Coli*			*Klebsiella pnemoniae*			*Pseudomonas aeruginosa*			*Acinetobacter baumannii*		
	Pre-COVID-19 Period (154)	COVID-19 Period (168)	*p*-Value	Pre-COVID-19 Period (10)	COVID-19 Period (15)	*p*-Value	Pre-COVID-19 Period (30)	COVID-19 Period (6)	*p*-Value	Pre-COVID-19 Period (36)	COVID-19 Period (8)	*p*-Value
Gentamicin	72.3	64.1	0.2886	65	57.1	0.3102	70	70	1.00	60	53.3	0.3922
Amikacin	60	53.8	0.4752	76	82.1	0.3856	100	100	1.00	39.5	20	0.0320
Cefotaxime	14	7.3	0.1652	23	7.1	0.0025	NR	NR	-	9.5	6.6	0.6133
Ciprofloxacin	27.3	14.4	0.0347	37.5	39.2	1.00	50	50	1.00	20	20	1.00
Imipenem	92	89.7	0.8056	46	67.8	0.0026	80	70	0.1412	100	88.6	0.0007
Tetracycline	ND	ND	-	ND	ND	-	NR	NR	-	36	13.3	0.0001
Cotrimoxazole	35	28.6	0.4486	32	32.1	1.00	NR	NR	-	34	13.3	0.0007
Nitrofurantoin	67.6	67.6	1.00	23	25	0.8686	23	30	0.3364	9.1	6.6	0.7953
Fosfomycin	98	96.5	1.00	56.7	46.4	0.2029	NR	NR	-	NR	NR	-
Piperacillin-Tazobactam	46	37.8	0.3159	61	67.8	0.3753	33.3	10	0.0003	16	6.6	0.0744
Ceftazidime	NR	NR	-	NR	NR	-	18	40	0.0010	NR	NR	-

NR—Not recommended according to CLSI; ND—Not performed as it belonged to either group C or O of CLSI; *p* < 0.05 was considered significant.

**Table 6:** Comparison of percentage antimicrobial susceptibility pattern of Gram positive bacteria in urine samples between pre-COVID-19 and COVID-19 period.

	Urinary Gram Positive Bacteria								
Antibiotics	*Staphylococcus aureus*			*Enterococcus* sp.			Coagulase Negative *Staphylococcus*		
	Pre-COVID-19 Period (26)	COVID-19 Period (17)	*p*-Value	Pre-COVID-19 Period (74)	COVID-19 Period (21)	*p*-Value	Pre-COVID-19 Period (2)	COVID-19 Period (5)	*p*-Value
Tetracycline	65	80	0.0261	60	100	0.0001	50	60	0.2007
Linezolid	100	100	1.00	100	100	1.00	100	100	1.00
Clindamycin	80	68.5	0.1042	NR	NR	-	50	80	0.0001
Cefoxitin	60	40	0.0071	NR	NR	-	50	60	0.2007
Erythromycin	43	25.7	0.0170	ND	ND	-	50	80	0.0001
Cotrimoxazole	17	20	0.7161	NR	NR		50	40	0.2007
Nitrofurantoin	100	100	1.00	70	48.8	0.0038	100	80	0.0001
High level gentamicin	NR	NR	-	54	48.8	0.5715	NR	NR	-
Ciprofloxacin	54.5	48.5	0.4792	50	48.8	1.00	50	80	0.0001

NR—Not recommended according to CLSI; ND—Not performed as it belonged to either group C or O of CLSI; *p* < 0.05 was considered significant.

## Data Availability

All relevant data are contained within the article.
